# Supplementary material for: Dynamics of multiple resistance mechanisms in plasma DNA during EGFR‐targeted therapies in non‐small cell lung cancer
Source: EMBO Mol Med. 2018 May 30;10(6):e7945. doi: 10.15252/emmm.201707945 (PMC5991591; doi:10.15252/emmm.201707945)
Supplement: Supplementary file 1 — Appendix [file EMMM-10-e7945-s001.pdf]

Appendix

Dynamics of multiple resistance mechanisms in plasma DNA during EGFR-targeted therapies in NSCLC

Dana Wai Yi TSUI, Muhammed MURTAZA, Alvin Seng Cheong WONG, Oscar M RUEDA, Chris G SMITH, Dineika CHANDRANANDA, Ross A SOO, Hong Liang LIM, Boon Cher GOH, Carlos CALDAS, Tim FORSHEW, Davina GALE, Wei LIU, James MORRIS, Francesco MARASS, Tim EISEN, Tan Min CHIN, Nitzan ROSENFELD

Appendix Methods

Appendix Figures S1-8

Appendix Tables S1-8

Appendix Methods

Sample collection and processing

Collected blood was spun down immediately (<1 hour) at 3,000 rpm for 20 min at the National University Hospital, Singapore. The plasma fraction was frozen and sent to Cancer Research UK Cambridge Institute, UK on dry ice. Thawed plasma samples were re-centrifuged at 14,400 rpm speed for 10 min to further separate any cellular portions and the supernatant before DNA was extracted from 0.8-2 ml of plasma using the Qiagen QIAamp Circulating Nucleic Acid Kit (Qiagen) according to manufacturer's protocol, except that we re-suspended carrier RNA to 1µg/ µL. The eluted DNA was stored at -20 °C until analysis.

Mutation identification and quantification by TAm-Seq and digital PCR

Plasma samples were analyzed by tagged-amplicon deep sequencing using the panel described previously (Forshew et al, 2012), with the addition of an amplicon that covers exon 18 of *EGFR* with the following target-specific primer sequences: forward primer TGGTGAGGGCTGAGGTG and reverse primer CCTGTGCCAGGGACCTTAC (using adapter sequences as previously specified). Mutation data were expressed as mutant allele fractions (AF). The principle of digital PCR for DNA quantification has been described previously (Vogelstein & Kinzler, 1999; Yung et al, 2009). Assays targeting the two hot-spot mutations on exon 19 (15-bp deletion) and exon 21 (L858R codon change 2753T>G) were performed as previously described (Yung et al, 2009). A TaqMan based assay targeting the T790M (2369C>T) resistance-conferring mutation in exon 20 was designed in this study. All digital PCR analysis was carried out on the BioMark system using 12.765 Digital Arrays (Fluidigm) following manufacturer's instructions and protocol. Data was analysed as previously described, except that for hotspot mutations (i.e. L858R and T790M), mutations were identified in a sample if non-reference AFs were above the respective background distribution at a confidence margin of 0.99 or greater in both replicates, instead of the threshold of 0.9995 or greater for other mutations. Mutant allele fractions were calculated based on weighted average (based on sequencing depth) across all replicates. The code used for

analysis of sequencing data has been licensed to Inivata and is not publicly available. The MAF measured by TAM-Seq and ddPCR strongly correlate with each other (Appendix Fig S8).

Sensitivity and specificity of the assays for Ex19 deletion and L858R were previously shown (Yung et al, 2009), and was reproducible in our data (details below). The T790M assay was evaluated by testing the assays in a serial 2-fold dilution of DNA extracted from a heterozygous cell line DNA (NCI-H1975, purchased from ATCC) carrying the mutation spiked in to healthy control plasma DNA. Quantification was linear over several orders of magnitude of dilution for all three assays (Appendix Fig. S7). Sensitivity of the T790M assays was tested using a plasma sample carrying T790M mutant sequences spiked in to healthy control plasma DNA such that the mutant allele frequency ranged from 0.1% to 5% (with 1-9 copies of mutant DNA spiked in to 748-955 copies of wild-type DNA). Mutant molecules were detected down to 0.1% expected allele frequency. Root mean square error (coefficient of variation) between expected and observed mutant allele frequency was 0.07 for allele frequencies at 0.06% (Appendix Fig S2b). The specificity of digital PCR assays for Exon 19 deletion, L858R and T790M were evaluated in a large volume of pooled healthy individual plasma samples. For the Exon 19 deletion and T790M assays, no mutant allele was detected out of >10,000 wild-type DNA molecules. For L858R, 3 false positive mutant allele were detected out of ~9500 wild-type DNA molecules, giving a false positive rate of 0.03% (Appendix Table S6). Every plasma sample was also quantified by digital PCR to estimate the total DNA copy number per ml plasma using a 65-bp assay targeting a region on the *RPP30* gene, a non-amplified region in the genome (Wang et al, 2010). The levels of total DNA may be affected by sample processing and extraction efficiency, which would in turn affect the sensitivity of detecting rare mutant allele. Therefore we measure the levels of total amplifiable copies of normal DNA in every sample as a quality check. The primer and probe sequences of all the digital PCR assays are summarized in Appendix Table S7. The mutant allele fractions were calculated based on the averaged values of digital PCR assay and TAm-Seq and reported in Appendix Dataset EV1.

Appendix Figures

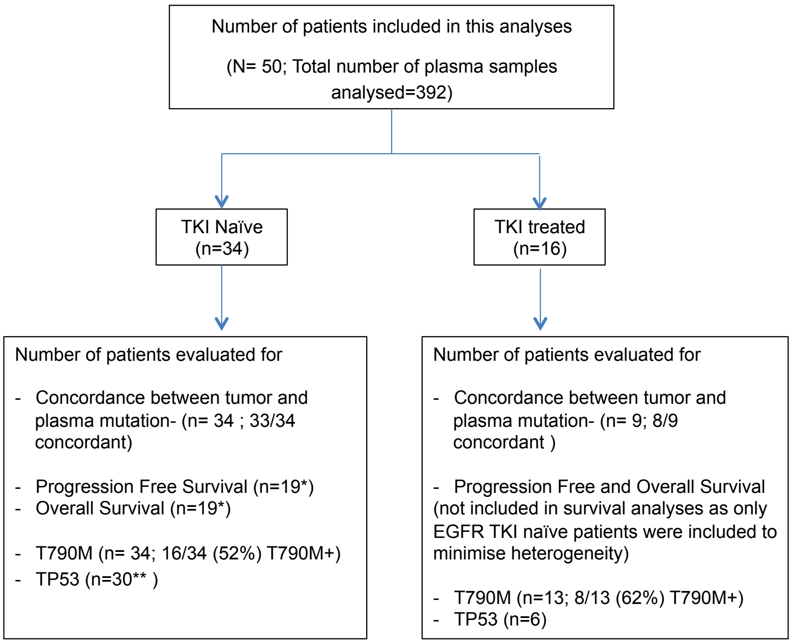

* N=19 (Only 19 pre-treatment samples were available for analyses. With initiation of EGFR TKI treatment, it is anticipated that mutation levels would drop. Hence only pre-treatment samples were used for survival analyses)
** N=30 (30 samples were used in this analyses. To increase power of analyses, samples drawn within 2 months of initiation of treatment were used).

Appendix Figure S1

Consort diagram showing the number of patients involved in different components of the study.

Of the 50 patients, 34 were TKI naïve and 16 were previously treated with TKI but progressed. We evaluated the concordance between tumor and plasma EGFR activating mutations and T790M status in both groups. Survival analysis was only performed in the TKI-naïve group.

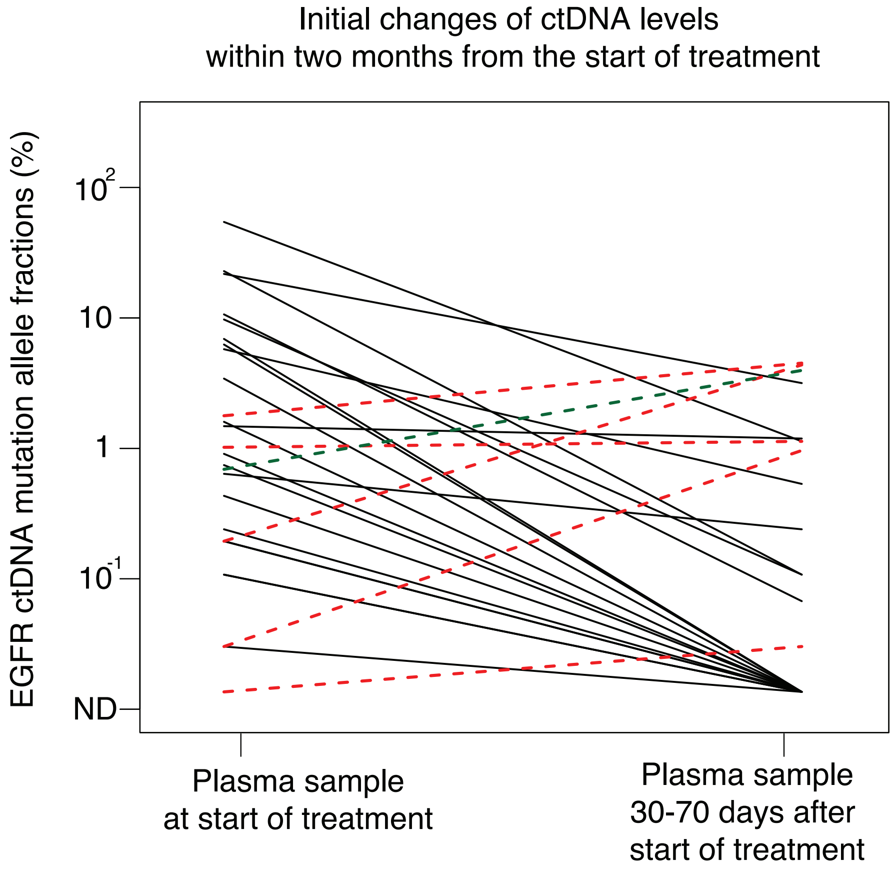

Appendix Figure S2

Initial changes of ctDNA levels in relation to radiographic responses

Initial change of *EGFR* mutant allele fractions in ctDNA of 26 patients where plasma samples were available before the start of treatment and at days 30-70 from start of treatment. Twenty-five patients had detectable ctDNA (non-zero) in either of the plasma samples. From these 25 patients, 24 responded to the treatment within the first 70 days, and 1 progressed. The black lines indicated a drop of ctDNA levels, red dotted lines indicated an increase for the patients who responded, and green dotted line indicated an increase for one patient who progressed, within this period of time.

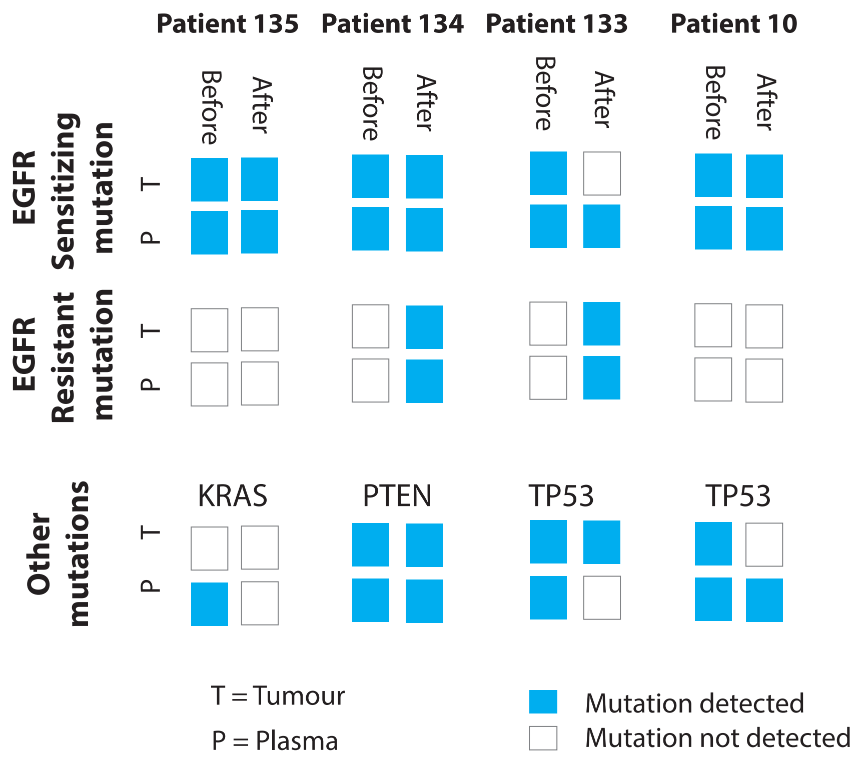

Appendix Figure S3

Concordance of mutation status before and after progression to EGFR-TKI

Summary of the mutation status in 4 patients where tumour and plasma samples were available before and after progression to EGFR-TKI. Status of EGFR sensitizing mutation (either L858R or Ex19 Del) and resistance-conferring (T790M) mutations are shown. Other mutations include TP53, KRAS and PTEN. The blue squares denote samples where mutations were detected, whereas the white square denote samples where mutations were not detected, before (pre-treatment) and after (closest to the time of progression) EGFR-TKI therapy.

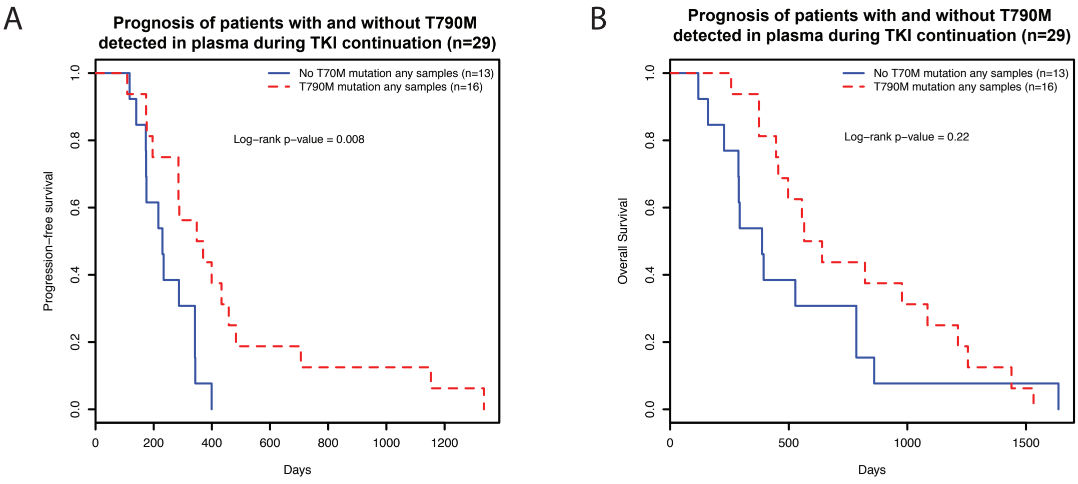

Appendix Figure S4

Prognostic value of the presence of T790M in plasma during TKI-continuation

(A and B) First-line TKI-treated patients that progressed with T790M detected in any of the plasma samples during TKI-continuation had significantly better progression-free survival (e, log-rank p-value = 0.008) and overall survival(log-rank p-value = 0.22), compared to patients who progressed with T790M detected in their plasma samples.

Appendix Figure S5

Patient 122

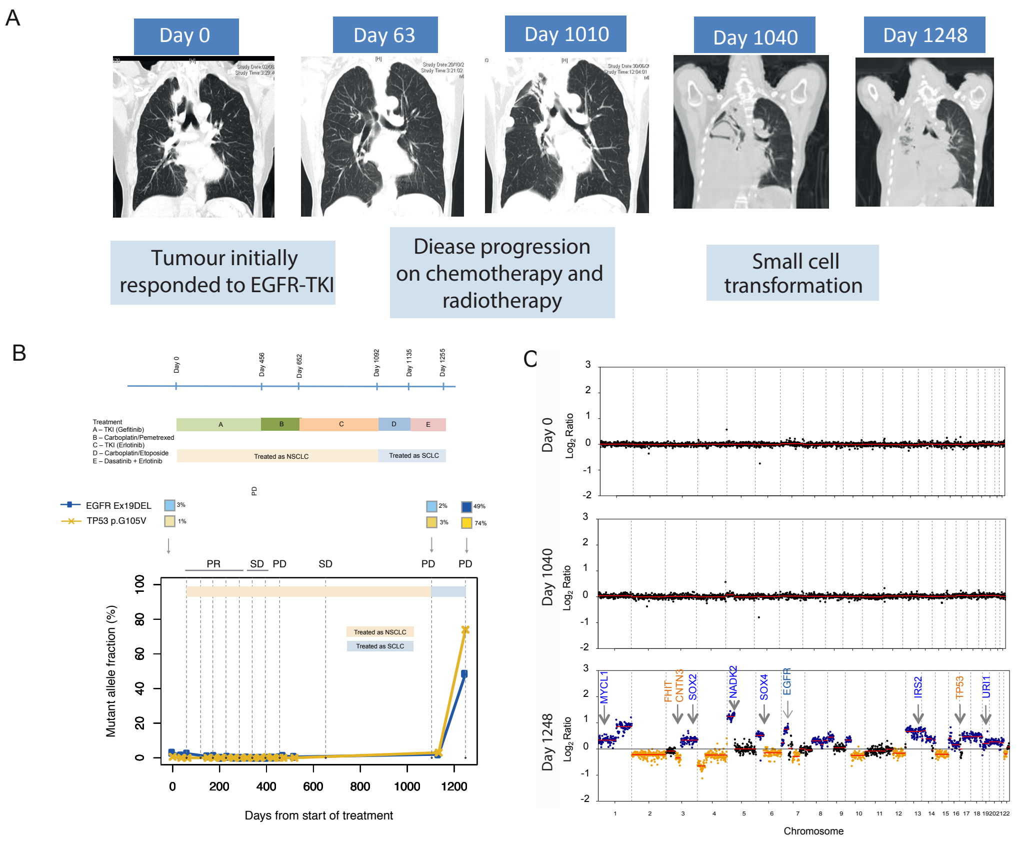

Global copy number changes and ctDNA dynamics in patient 122 before and after histological transformation to SCLC

A) Imaging scans showing radiographic records of clinical responses and progression before and after SCLC transformation (Day 1040). B) Dynamics of somatic point mutations in longitudinal plasma samples collected during the treatment. C) Global copy number profiles in selected plasma samples collected during the treatment before and after SCLC transformation. CNV events that were significantly identified and coincide with literature-reported SCLC events are denoted in colours: blue for gain and orange for loss.

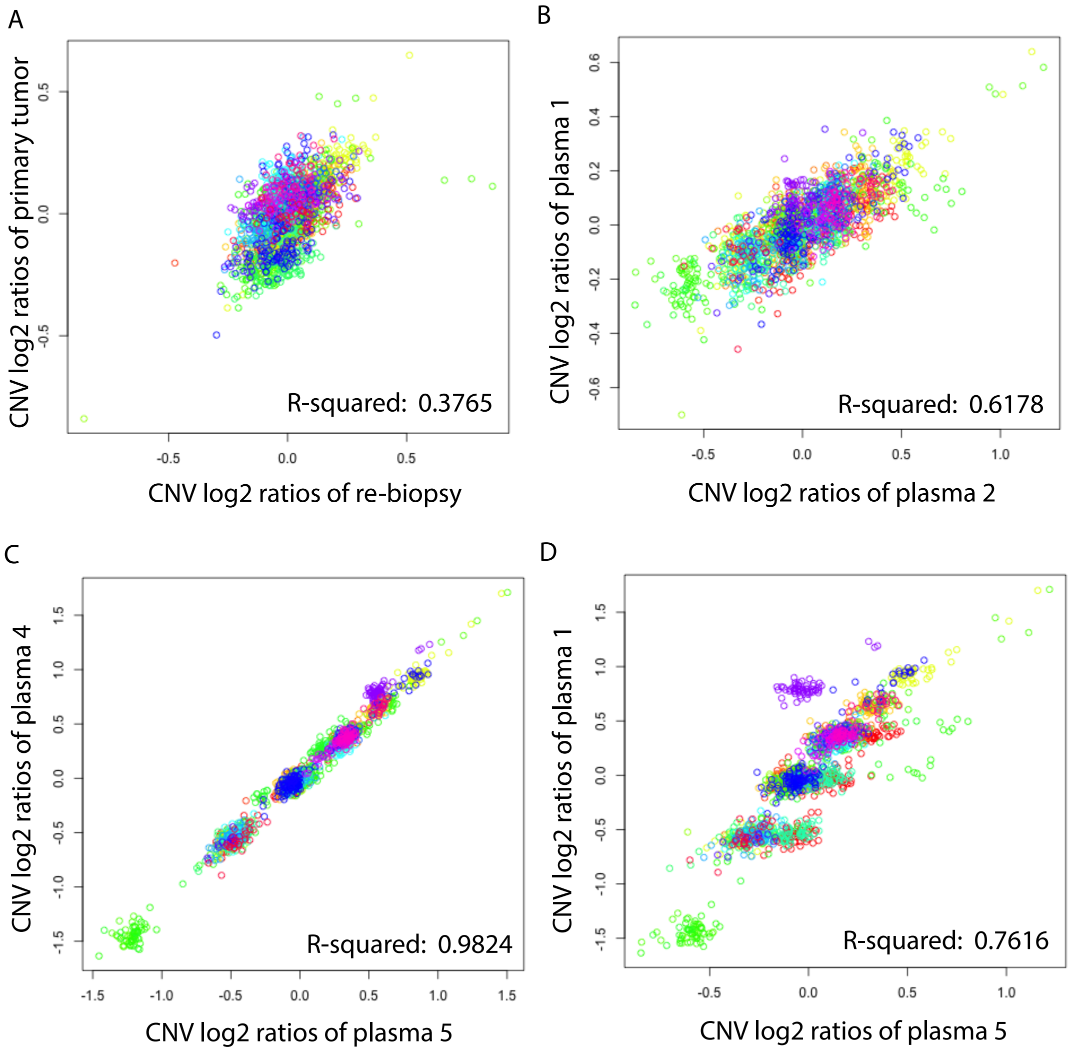

Appendix Figure S6

Correlation between the segmented log2 ratio of different plasma samples of patient 218. Relationship between A) the tumour samples at baseline and relapse, B) plasma 1 (day 78) and 2 (day 329) before and after sequential treatment of chemotherapy carboplatin with alimta, then docetaxel; C) plasma 4 (day 500) and 5 (day 534), after progression on combination treatment of cisplatin and etoposide; D) before and after confirmation of SCLC transformation, between plasma 1 (day 78) and plasma 5 (day 534). The adjusted R-square for the correlation are shown.

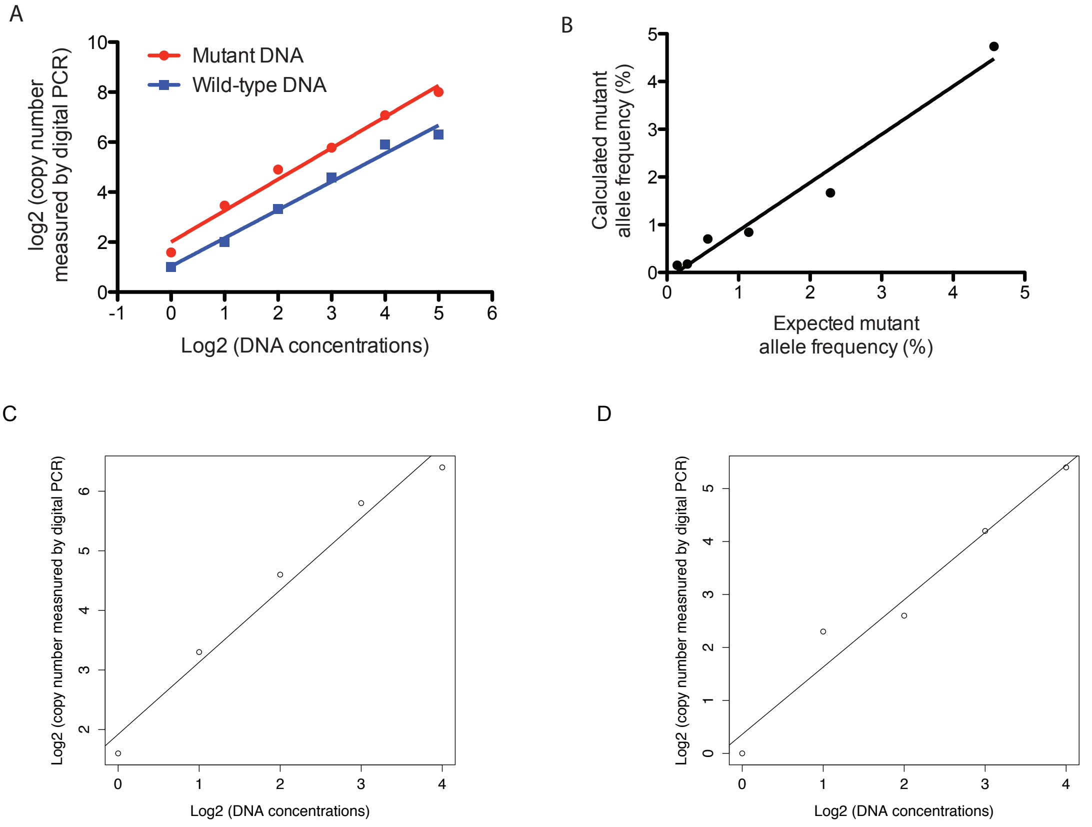

Appendix Figure S7

Digital PCR assays evaluation

A-D) Quantitative performance of digital PCR assay in serially diluted mutant and wild-type DNA

A. Quantitative performance of digital PCR analysis for T790M mutation. A serial 2-fold dilution of cell line DNA carrying a T790M mutation was prepared by dilution into water and analyzed by digital PCR. Quantification by digital PCR (log2 of copy number measured by digital PCR) was linear over >2 orders of magnitude of dilution.

B. A plasma sample carrying T790M mutant sequences was mixed with wildtype plasma DNA such that the mutant allele frequency ranged from 0.1% to 5%. (with 1-9 copies of mutant DNA spiked in to 748-955 copies of wild-type DNA). The mixtures were analyzed with digital PCR. Mutant molecules were detected down to 0.1% expected allele frequency. Root mean square

error (coefficient of variation) between expected and observed mutant allele frequency was 0.07 for allele frequencies at 0.06%.

C-D. Quantitative performance of digital PCR analysis for (C) L858R and (D) Exon 19 Deletion mutations. A serial 2-fold dilution of cell line DNA carrying the respective mutations were prepared by dilution into water and analyzed by digital PCR. Quantification by digital PCR (log2 of copy number measured by digital PCR) was linear over >2 orders of magnitude of dilution, in agreement with the linearity of quantification of these assays previously described (Yung et al, 2009).

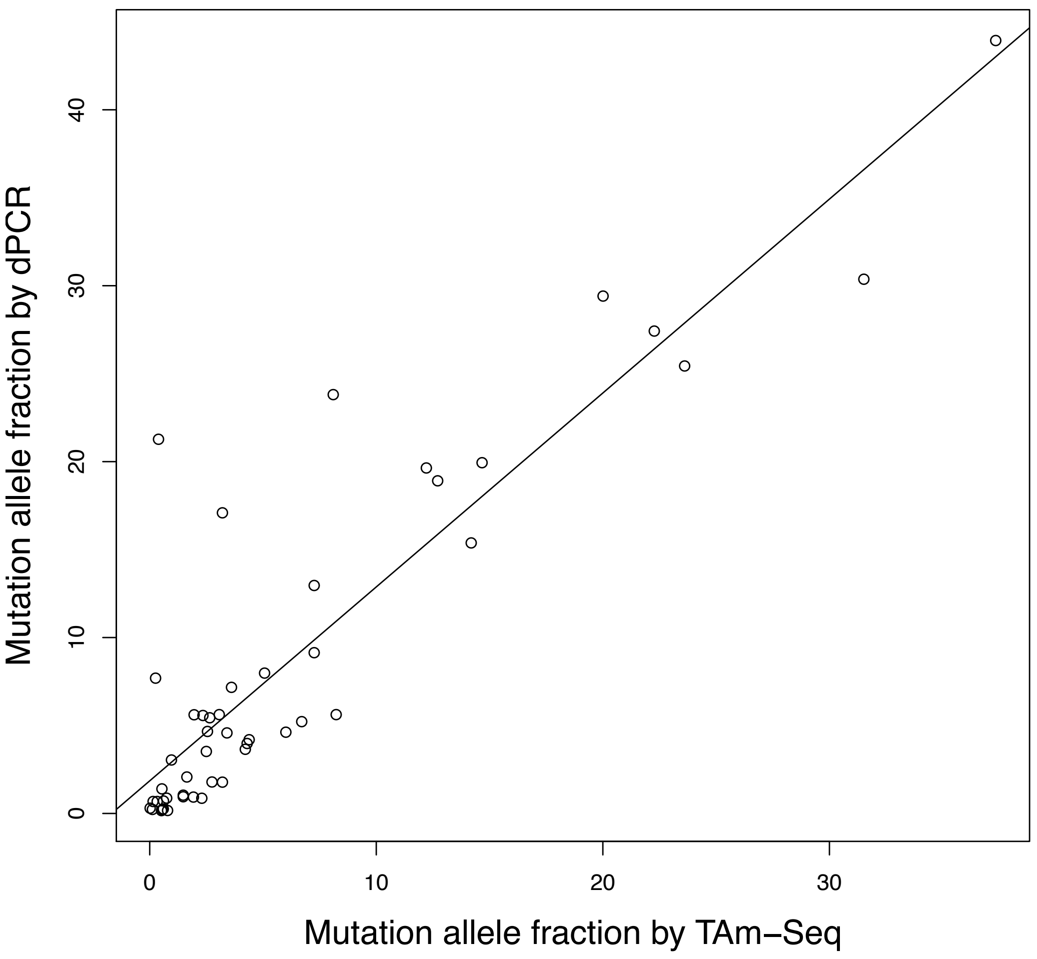

Appendix Figure S8

Correlation of mutation allele fraction measured by TAm-Seq and digital PCR (dPCR)

(E) There are 49 data-points where both measurements of TAm-Seq and dPCR are non-zero. The mutation allele fractions of L858R and T790M measured by TAM-Seq and digital PCR (dPCR) are significantly correlated with each other (p-value <2e-16, R-squared = 0.7982). The root mean square (RMS) relative error between the two measurements was 26% for mutant allele fraction >1%.

Appendix Tables:

Patient study number	EGFR detected in tumor (hospital record)			Additional EGFR mutations detected in plasma		Other cancer mutations detected			
	Mutation details	Mutant AF* in first available plasma (%)	EGFR status in tumor and plasma agreed	Mutations details	Mutant AF* in first plasma (%)	In plasma collected during treatment	In matched normal DNA (if available)	In pre-treatment plasma (if available)	In pre-treatment tumour (if available)
**TKI-naïve**									
**3**	ND (Wild type)	ND	Yes		ND	-		-	-
**5**	ND (Wild type)	ND	Yes		ND	-		-	-
**6**	Ex19 DEL del 747-E749.	2.10	Yes			-		-	-
**8**	Ex19 DEL del E746-A750	0.54	Yes			-		-	-
**10**	p.L858R	1.99	Yes	p.L1038V	53.70	TP53 p.E258K	ND	Yes	Yes
				p.V738F	49.66				
**12**	p.L858R	0.92	Yes			TP53 p.R267W	ND	Yes	Yes
**101**	Ex19 DEL del L747-A750insP	0.28	Yes	p.A864T	ND^	-			-
**103**	Ex19 DEL del L747-E749, A750P	2.51	Yes			-			-
**104**	p.G719S	10.20	Yes			BRAF p.G593V	ND	NA	ND
				p.N700T	2.30				
				p.Q701K	2.00				
				p.A698D	0.80				
				p.L861Q	13.90				
				p.L858R	0.05				
**105**	Ex19 DEL del E746-A750	4.34	Yes			-			-
**106**	p.L858R	ND^	Yes			TP53 p.P223A, PIK3CA p.E545A	ND	Yes, Yes	ND, ND
**107**	Ex19 DEL K745-E746insIPVAIK	0.06	Yes	p.A767P	ND^	TP53 p.C176F ,PIK3CA p.E545A	ND	NA	Yes, ND
**108**	p.L858R	23.81	Yes			TP53 p.S241C	ND	Yes	NA
**109**	p.L858R	30.37	Yes			TP53 chr17:7578176 C>A	ND	Yes	NA
**112**	p.L858R	0.07	Yes			-			-
**113**	Ex19 DEL del E746-A750	3.07	Yes	p.D994E	ND^	BRAF p.G593V, PIK3CA p.T1061K, TP53 p.Y103X	ND	NA	NA
**115**	p.L858R	15.38	Yes			-		-	-
**116**	Ex19 DEL del E746-A750	3.39	Yes			-		-	-
				p.L858R	0.68				
**117**	Ex19 DEL del E746-T751	65.65	Yes			TP53 p.R248Q, PIK3CA p.T1061K	ND	Yes, ND	Yes, ND
**119**	Ex19 DEL del E746-A750	0.64	Yes			-		-	-
**120**	p.L858R	12.96	Yes			TP53 p.H179R	ND	Yes	NA
				Ex19 DEL	0.20				
**122**	Ex19 DEL del E746-A750	2.94	Yes			TP53 p.G105V	ND	Yes	Yes
**123**	Ex19 DEL del T751-I759	0.27	Yes			PIK3CA p.T1052K	ND	Yes	ND
**124**	p.L858R	1.01	Yes			TP53 p.L257R	ND	Yes	NA
				Ex19 DEL	0.15				
**125**	p.L858R	5.22	Yes			TP53 p.P152L	ND	Yes	Yes
**126**	Ex19 DEL del 746-A750	ND^	Yes			PTEN p.V317G	ND	NA	ND
**127**	Ex19 DEL del 746-A750	10.96	Yes			TP53 p.P87L, TP53 p.Q331X, TP53 p.Y220C	ND	Yes, Yes, Yes	ND, Yes Yes
**128**	Ex19 DEL del 746-A750	0.17	Yes			-			-
**129**	Ex19 DEL del 746-A750	0.30	Yes			PTEN p.D92H	ND	ND^	Yes
**130**	p.L858R	2.75	Yes			TP53 p.I195T	ND	Yes	Yes
**131**	G719S (hospital record)/p.L861Q	ND/1.00	No			TP53 p.P87T, TP53 p.R342X	ND	NA, NA	ND, Yes
**133**	Ex19 DEL del L747-P753insS	ND	No			TP53 p.Q331X	ND	Yes	Yes
				p.L858R	0.28				Yes
**134**	p.L858R	1.56	Yes			PTEN p.Q171X	ND	Yes	Yes
**135**	264delTTAAGAGAAGCAACATCTCC(	ND^	Yes	p.L858R	0.17	KRAS p.G12A, KRAS p.G13D	ND	NA	ND, ND
**TKI-treated**									
**11**	Ex19 DEL del 746-A750	29.65	Yes			PTEN p.S227F, TP53 p.R248Q, TP53 p.P87T, PIK3CA p.H1060Q	ND	ND^,Yes,ND^,ND^	NA
**201**	NA[$]	-		Ex19 DEL	1.87	-		-	-
				p.L858R	0.13				
**202**	p.L858R	27.42	Yes			TP53 p.R273H, TP53 p.T253P	ND	NA	NA
**203**	p.G719A	ND^	Yes	p.T783P	0.72	-			-
**204**	NA	-		p.L858R	25.44	-		-	-
				p.A767P	22.30				
**205**	NA	-		p.L858R	21.27	-			-
**206**	p.L858R	4.94	Yes			-			-
**207**	Ex19 DEL	0.28	Yes			-			-
**208**	NA	-		Ex19 DEL	3.01	TP53 p.P87T, TP53 p.P177L, TP53 p.P72S	ND	NA	NA
**209**	NA	-		Ex19 DEL	9.38	TP53 p.Y163C	ND	NA	NA
**210**	Ex19 DEL del 746-A750	0.06	Yes			-		-	-
**211**	NA	-		Ex19 DEL	1.45	-		-	-
**212**	NA	-		Ex19 DEL	2.11	-			-
**223**	Ex19 DEL del 746-A750	-				TP53 p.G154S, PIK3CA p.E545K	ND	Yes	NA
**220**	Ex19 DELdel E746-A750	ND^	Yes			TP53 p.Y103X	ND	Yes	NA
**218**	L858R	0.3	Yes			TP53 p.R175H, TP53 p.A307A	ND	Yes	Yes

^Detected in follow up samples
*** AF = Allele Fraction**
NA = Materials not available
NA[$] = Patients enrolled into the trial based on the criteria that they had progressed on previous TKI treatment
ND = Not Detected

Appendix Table S1. Mutation profiles of the 47 patients

Table summarizing the mutation profiles of the 47 patients. The EGFR mutations status in tumor was obtained based on hospital record or TAm-Seq/digital PCR analysis if tumor materials were available. The status of the tumor mutations in the first available plasma was shown with the mutant allele fractions (AF) indicated in percentage (%), or ND (not detected), and mutations that were detected in subsequent follow-up plasma were indicated with ND^. Additional *EGFR* mutations detected in plasma during treatment and their AF in first available plasma were also summarized. Mutations other than *EGFR* that were detected during treatment were listed, with indication of whether they were detected in matched normal DNA, pre-treatment plasma, or pre-treatment tumor, if available respectively.

Patient study number	First CT scan results on 60 days	First available plasma sample		Plasma sample collected after 30-70 days	
		Days from start of treatment	EGFR ctDNA levels (AF in %)	Days from start of treatment	EGFR ctDNA levels (AF in %)
117	SD	-1	65.7	35	2.3
109	PR	0	31.0	63	0.3
11	SD	-5	29.7	61	5.6
108	PR	0	16.0	66	0.2
115	PR	0	14.8	63	0.3
127	PR	0	11.0	63	0.0
120	PR	0	10.1	63	0.0
209	SD	7	9.4	60	1.2
125	PR	0	6.0	34	0.0
116	PR	-1	3.4	63	7.6
113	SD	6	3.1	68	0.0
122	PR	0	2.9	63	2.4
6	CR	-2	2.1	62	2.3
201	SD	-5	1.9	67	0.0
134	PR	0	1.6	64	0.0
211	PD	0	1.5	35	6.8
130	PR	0	1.4	63	0.6
10	PR	-7	1.0	63	0.0
119	PR	0	0.6	63	0.0
8	PR	-12	0.5	70	7.4
124	PR	0	0.5	56	0.0
12	PR	-18	0.5	63	0.0
129	SD	0	0.3	62	0.0
123	SD	0	0.3	62	0.0
133	PR	0	0.1	64	0.0
210	SD	0	0.1	63	2.0
106	SD	0	0.0	66	0.1
131	PR	7	0.0	70	0.0
101	SD	33	-	89	-
103	PR	21	-	49	-
104	PR	35	-	35	-
105	SD	68	-	68	-
107	PR	34	-	64	-
112	SD	35	-	35	-
126	SD	33	-	90	-
128	PR	35	-	63	-
135	PR	35	-	63	-
202	SD	29	-	62	-
203	SD	36	-	64	-
204	PD	0	-	0	-
205	PD	34	-	34	-
206	SD	41	-	41	-
207	PD	61	-	61	-
208	PD	10	-	10	-
212	SD	34	-	60	-
223	SD	0	-	354	-

Appendix Table S2. Initial changes of EGFR ctDNA levels and clinical response

Table summarizing the initial responses (represented by CT scan results) of the 45 patients who harbour EGFR activating mutations, the collection days (from start of treatment) of their first available plasma and samples collected after 30-70 days (closet to the date when initial CT scan results). The *EGFR* ctDNA levels of each sample were shown, for 28 patients where the first available plasma samples were collected within 7 days of start of treatment, and there was a sample within 30-70 days from start of treatment. CT scan results were represented by SD, stable disease, PR, partial response, CR, complete response, PD, progressive disease.

Patient study number	Sample	Date from start of sample colleciton / start of treatment	CT data	EGFR activating mutation allele fraction %		EGFR resistance-conferring mutation (T790M) allele fraction %		Additional mutation (1)	Protein change (1)	Mutation details (1)	Mutant allele fraction % (1)	Additional mutation (2)	Protein change (2)	Mutation details (2)	Mutant allele fraction % (2)
10	Pre-treatment tumour			L858R	39.00	T790M	0.00	TP53	p.E258K	17:7577509_C>T	60.40				
10	Plasma 1	-7		L858R	1.99	T790M	0.00	TP53	p.E258K	17:7577509_C>T	0.42				
10	Plasma 2	35		L858R	0.00	T790M	NA	TP53	p.E258K	17:7577509_C>T	0.00				
10	Plasma 3	63	PR	L858R	0.00	T790M	NA	TP53	p.E258K	17:7577509_C>T	0.00				
10	Plasma 4	118		L858R	0.82	T790M	NA	TP53	p.E258K	17:7577509_C>T	0.00				
10	Plasma 5	146		L858R	0.69	T790M	0.00	TP53	p.E258K	17:7577509_C>T	1.95				
10	Plasma 6	174	PD	L858R	1.40	T790M	0.00	TP53	p.E258K	17:7577509_C>T	1.35				
10	Post-treatment tumour			L858R	3.40	T790M	0.00	TP53	p.E258K	17:7577509_C>T	0.00				
133	Pre-treatment tumour			L858R	2.56	T790M	0.00	TP53	p.Q331X	17:7576855_G>A	8.30				
133	Plasma 1	0		L858R	0.28	T790M	0.00	TP53	p.Q331X	17:7576855_G>A	2.34				
133	Plasma 2	36		L858R	0.00	T790M	0.00	TP53	p.Q331X	17:7576855_G>A	0.00				
133	Plasma 3	64	PR	L858R	0.00	T790M	0.00	TP53	p.Q331X	17:7576855_G>A	0.00				
133	Plasma 4	90		L858R	0.00	T790M	0.81	TP53	p.Q331X	17:7576855_G>A	0.00				
133	Plasma 5	111	PD	L858R	4.00	T790M	0.39	TP53	p.Q331X	17:7576855_G>A	0.00				
133	Post-treatment tumour			L858R	0.00	T790M	5.60	TP53	p.Q331X	17:7576855_G>A	11.70				
134	Pre-treatment tumour			L858R	36.40	T790M	0.00	PTEN	p.Q171X	10:89711893_C>T	77.75				
134	Plasma 1	0		L858R	1.56	T790M	0.00	PTEN	p.Q171X	10:89711893_C>T	2.34				
134	Plasma 2	36		L858R	0	T790M	0.00	PTEN	p.Q171X	10:89711893_C>T	0.19				
134	Plasma 3	64	PR	L858R	0	T790M	0.00	PTEN	p.Q171X	10:89711893_C>T	0.69				
134	Plasma 4	92		L858R	2.5	T790M	0.29	PTEN	p.Q171X	10:89711893_C>T	0.00				
134	Plasma 5	117		L858R	0.22	T790M	0.34	PTEN	p.Q171X	10:89711893_C>T	1.06				
134	Plasma 6	148		L858R	0	T790M	0.42	PTEN	p.Q171X	10:89711893_C>T	0.70				
134	Plasma 7	176	PD	L858R	0	T790M	0.18	PTEN	p.Q171X	10:89711893_C>T	0.49				
134	Plasma 8	211		L858R	2.74	T790M	0.25	PTEN	p.Q171X	10:89711893_C>T	3.30				
134	Plasma 9	239		L858R	0.5	T790M	0.00	PTEN	p.Q171X	10:89711893_C>T	1.34				
134	Post-treatment tumour			L858R	23.80	T790M	7.49	PTEN	p.Q171X	10:89711893_C>T	41.30				
134	Plasma 10	260		L858R	0	T790M	0.00	PTEN	p.Q171X	10:89711893_C>T	0.44				
135	Pre-treatment tumour			Ex19 Del	50.00	T790M	0.00	KRAS	p.G13D	12:25398281_C>T	0.00	KRAS	p.G12A	12:25398284_C>G	0.00
135	Plasma 1	35		Ex19 Del	0.00	T790M	0.00	KRAS	p.G13D	12:25398281_C>T	1.15	KRAS	p.G12A	12:25398284_C>G	1.40
135	Plasma 2	63	PR	Ex19 Del	0.59	T790M	0.00	KRAS	p.G13D	12:25398281_C>T	0.00	KRAS	p.G12A	12:25398284_C>G	0.00
135	Plasma 3	91		Ex19 Del	0.52	T790M	0.00	KRAS	p.G13D	12:25398281_C>T	0.28	KRAS	p.G12A	12:25398284_C>G	0.00
135	Plasma 4	121	PD day 175	Ex19 Del	1.39	T790M	0.00	KRAS	p.G13D	12:25398281_C>T	0.00	KRAS	p.G12A	12:25398284_C>G	0.00
135	Post-treatment tumour			Ex19 Del	66.70	T790M	0.00	KRAS	p.G13D	12:25398281_C>T	0.00	KRAS	p.G12A	12:25398284_C>G	0.00

Appendix Table S3. Mutation profiles in 4 patients with pre- and post-treatment tumor and plasma samples

Mutation data of 4 patients with pre- and post-treatment tumor and plasma samples are shown. Data are shown according to the days from start of treatment. Radiographic responses were assessed by CT imaging. PR: partial responses; PD: Progressive disease.

Patient study number	Day from start of treatment when first plasma sample taken	Time to events in days (progression)♯	Time to events in days (Survival)♯	First plasma sample EGFR activating mutation alelle fraction %	First plasma sample EGFR activating mutation copies/ml	First plasma sample most abundant mutation alelle fraction %	First plasma sample most abundant mutation copies/ml	Classification of mutation titres	Tumor measurement via RECIST (mm)	Median tumor volume of respective groups	Presence of extra-thoracic disease in addition to intra-thoracic disease
109	0	202	374	30.4	4588.6	30.4	4588.6	High	80	80	No
115	0	175	304	15.4	83.5	15.4	83.5	High	38		Yes
120	0	275	(275)	13.0	662.1	13.0	662.1	High	66		No
125	0	433	(433)	5.2	200.5	5.2	200.5	High	81		No
127	0	174	(174)	11.0	1362.1	11.0	1362.1	High	93		No
6	2 days before start of treatment	427	785	2.1	53.3	2.1	53.3	Intermediate	42	42	No
10	7 days before start of treatment	174	293	2.0	62.4	2.0	62.4	Intermediate	25		No
12	18 days before start of treatment	399	565	0.9	128.6	0.9	128.6	Intermediate	42		No
116	1 day before start of treatment	289	399	3.4	66.3	3.4	66.3	Intermediate	53		No
119	0	284	397	0.6	90.7	0.6	90.7	Intermediate	15		No
122	0	458	(519)	2.9	350.4	2.9	350.4	Intermediate	49		No
124	0	216	300	1.0	51.6	8.5	434.3	Intermediate	54		Yes
130	0	233	(301)	2.8	104.6	6.4	243.5	Intermediate	31		No
134	0	176	(211)	1.6	77.8	2.2	110.0	Intermediate	23		No
8	12 days before start of treatment	706	(706)	0.5	38.9	0.5	38.9	Low	45	17	No
106	0	846	(846)	0.0	0.0	9.8	1803.8	Low	54		No
123	0	230	(230)	0.3	12.5	2.0	92.8	Low	17		No
129	0	(1042)	(1042)	0.3	6.3	0.3	6.3	Low	15		Yes
133	0	109	271	0.3	37.0	2.4	316.9	Low	10		No

♯ censored days in parenthesis

Note: Patient 108 and 117 were TKI-naïve but have withdrawn from the study during their treatment, hence not included in the prognosis analysis.

Appendix Table S4. Survival, mutational and tumor volume data of 19 TKI-naïve patients with available pre-treatment cfDNA samples

Survival data of 19 TKI-naïve patients who had cfDNA detected in first available plasma before treatment initiation. CfDNA levels in first plasma samples were estimated based on EGFR activating mutations as mutant allele fractions (MAF) and copies/ml plasma respectively in columns 4 and 5. When multiple mutations were present, cfDNA levels were estimated based on the most abundant mutations (i.e. where MAF or copies/ml plasma were highest) in columns 6 and 7, respectively. Mutation levels were classified as low, intermediate and high based on below 25% quartile, intermediate and above 75% quartile. Clinical information about the tumors such as RECIST measurements, median volume and the presence of extra-thoracic disease were shown.

Patient study number	Detection of EGFR T790M in plasma			Time to events in days (censored days in parenthesis)	
	Maximal AF during treatment (%)	Time from start of treatment to detection (days)♯	Lead time from detection to disease progression (days)♯	Progression	Survival
**TKI-naïve**					
**6**	ND			343	785
**8**	0.18	70	636	706	1213
**10**	ND			174	293
**12**	0.6	18 days before start of treatment	417	399	565
**101**	6.22	873	280	1153	1439
**103**	4.66	189	111	285	821
**104**	ND			117	119
**105**	ND			342	1637
**106**	0.31	454	392	483	1085
**107**	ND			140	159
**109**	0.18	35	167	196	374
**112**	ND			342	785
**113**	0.64	545	918	1335	1532
**115**	ND			173	288
**116**	0.46	35	254	285	555
**119**	ND			287	394
**120**	3.98	203	72	288	446
**122**	0.61	37	421	458	1255
**123**	ND			230	528
**124**	ND			216	289
**125**	1.77	230	203	433	497
**126**	2.47	202	146	348	640
**127**	0.75	119	55	174	456
**128**	ND			399	860
**130**	ND			234	387
**131**	1.56	265	50	370	977
**133**	0.81	90	19	109	257
**134**	0.42	92	84	176	374
**135**	ND			175	227

*** AF = Allele Fraction**
♯Number in parenthesis are right-censored
ND = Not Detected

Note: Patient 108 and 117 were TKI-naïve but have withdrawn from the study during their treatment, hence not included in the prognosis analysis.

Appendix Table S5. Detection of T790M in plasma samples and relationship with prognosis

Table summarising whether *EGFR* T790M was detected in any plasma samples during treatment, and if detected, the days of detection from start of treatment and from time of onset of disease progression, respectively, were shown. The survival data were shown as time to events in days (progression or overall survival).

Assays	Poisson-corrected copy number of mutant DNA	Poisson-corrected copy number of wild-type DNA	False positive rate
EGFR Ex 19 deletion	0	577	
EGFR Ex 19 deletion	0	2416	
EGFR Ex 19 deletion	0	114	
EGFR Ex 19 deletion	0	103	
EGFR Ex 19 deletion	0	481	
EGFR Ex 19 deletion	0	448	
EGFR Ex 19 deletion	0	536	
EGFR Ex 19 deletion	0	461	
EGFR Ex 19 deletion	0	244	
EGFR Ex 19 deletion	0	272	
EGFR Ex 19 deletion	0	278	
EGFR Ex 19 deletion	0	258	
EGFR Ex 19 deletion	0	495	
EGFR Ex 19 deletion	0	468	
EGFR Ex 19 deletion	0	394	
EGFR Ex 19 deletion	0	427	
EGFR Ex 19 deletion	0	454	
EGFR Ex 19 deletion	0	506	
EGFR Ex 19 deletion	0	540	
EGFR Ex 19 deletion	0	605	
Total	0	10077	0.00%

EGFR L858R	0	457	
EGFR L858R	0	360	
EGFR L858R	1	386	
EGFR L858R	0	635	
EGFR L858R	0	550	
EGFR L858R	0	434	
EGFR L858R	0	628	
EGFR L858R	0	550	
EGFR L858R	0	299	
EGFR L858R	0	198	
EGFR L858R	1	157	
EGFR L858R	0	386	
EGFR L858R	0	357	
EGFR L858R	0	366	
EGFR L858R	0	508	
EGFR L858R	0	514	
EGFR L858R	0	107	
EGFR L858R	0	110	
EGFR L858R	0	105	
EGFR L858R	0	117	
EGFR L858R	0	608	
EGFR L858R	0	487	
EGFR L858R	0	195	
EGFR L858R	1	168	
EGFR L858R	0	278	
EGFR L858R	0	296	
EGFR L858R	0	128	
EGFR L858R	0	141	
Total	3	9525	0.03%

Assays	Poisson-corrected copy number of mutant DNA	Poisson-corrected copy number of wild-type DNA	False positive rate
EGFR T790M	0	55	
EGFR T790M	0	89	
EGFR T790M	0	45	
EGFR T790M	0	60	
EGFR T790M	0	170	
EGFR T790M	0	156	
EGFR T790M	0	139	
EGFR T790M	0	168	
EGFR T790M	0	119	
EGFR T790M	0	95	
EGFR T790M	0	103	
EGFR T790M	0	114	
EGFR T790M	0	132	
EGFR T790M	0	82	
EGFR T790M	0	5104	
EGFR T790M	0	233	
EGFR T790M	0	579	
EGFR T790M	0	127	
EGFR T790M	0	94	
EGFR T790M	0	91	
EGFR T790M	0	91	
EGFR T790M	0	78	
EGFR T790M	0	489	
EGFR T790M	0	481	
EGFR T790M	0	158	
EGFR T790M	0	113	
EGFR T790M	0	143	
EGFR T790M	0	102	
EGFR T790M	0	119	
EGFR T790M	0	105	
EGFR T790M	0	81	
EGFR T790M	0	72	
EGFR T790M	0	76	
EGFR T790M	0	24	
EGFR T790M	0	35	
EGFR T790M	0	126	
EGFR T790M	0	60	
EGFR T790M	0	66	
EGFR T790M	0	70	
EGFR T790M	0	79	
EGFR T790M	0	73	
EGFR T790M	0	21	
Total	0	10417	0.00%

Appendix Table S6. Specificity of the digital PCR assays

The specificity of digital PCR assays for Exon 19 deletion, L858R and T790M were tested in healthy plasma samples. No positive detection of mutant allele was seen for the Exon 19 deletion and T790M assay in >10,000 wild-type DNA molecules. For L858R, 3 false positive detections were observed out of ~9500 wild-type DNA molecules, giving a false positive rate of 0.03%.

Gene	Protein change	Mutation position	Mutation details	Amplicon position	Forward primer	Reverse primer	Amlplicon length
EGFR	p.I744_E749>LKR	chr7:55242460-55242477	15bp inframe deletion	chr7:55242438-55242541	GAAAGTTAAAATTCCCGTCGCTAT	ACCCCCACACAGCAAAGC	104bp
EGFR	p.L858R	chr7:55259515	T>G	chr7:55259484-55259561	CCGCAGCATGTCAAGATCAC	TCCTTCTGCATGGTATTCTTTCTCT	78bp
EGFR	p.T790M	chr7:55249071	C>T	chr7:55249038-55249122	GCATCTGCCTCACCTCCA	TCTTTGTGTTCCCGGACATAG	85bp
RPP30	-	-	-	chr10:92631758-92631822	AGATTTGGACCTGCGAGCG	GAGCGGCTGTCTCCACAAGT	65bp

Gene	Protein change	Mutation position	Mutation details	Probe (Mutant)	Probe (Wild-type)	Tm
EGFR	p.I744_E749>LKR	chr7:55242460-55242477	15bp inframe deletion	ACATCGAGGATTTCCTTGT	AATTAAGAGAAGCAACATC	55C
EGFR	p.L858R	chr7:55259515	T>G	TTGGCCCGCCCAA	TTGGCCAGCCCAA	56C
EGFR	p.T790M	chr7:55249071	C>T	TCATCATGCAGCTCAT	TCATCACGCAGCTCA	60C
RPP30	-	-	-	-	TTCTGACCTGAAGGCTCTGCGCG	60C

Appendix Table S7. Primer and probe sequences of digital PCR assays

Table summarizing the primer and probe sequences of digital PCR assays for *EGFR* Exon 19 deletion, L858R and T790M mutations, as well as for the *RPP30* gene.

References:

Forshew T, Murtaza M, Parkinson C, Gale D, Tsui DW, Kaper F, Dawson SJ, Piskorz AM, Jimenez-Linan M, Bentley D, Hadfield J, May AP, Caldas C, Brenton JD, Rosenfeld N (2012) Noninvasive identification and monitoring of cancer mutations by targeted deep sequencing of plasma DNA. Sci Transl Med 4**:** 136ra168

Vogelstein B, Kinzler KW (1999) Digital PCR. Proc Natl Acad Sci U S A 96**:** 9236-9241

Wang J, Ramakrishnan R, Tang Z, Fan W, Kluge A, Dowlati A, Jones RC, Ma PC (2010) Quantifying EGFR alterations in the lung cancer genome with nanofluidic digital PCR arrays. Clin Chem 56**:** 623-632

Yung TK, Chan KC, Mok TS, Tong J, To KF, Lo YM (2009) Single-molecule detection of epidermal growth factor receptor mutations in plasma by microfluidics digital PCR in non-small cell lung cancer patients. Clin Cancer Res 15**:** 2076-2084
